# Herpes Simplex Virus-Type1 (HSV-1) Impairs DNA Repair in Cortical Neurons

**DOI:** 10.3389/fnagi.2016.00242

**Published:** 2016-10-18

**Authors:** Giovanna De Chiara, Mauro Racaniello, Cristiana Mollinari, Maria Elena Marcocci, Giorgia Aversa, Alessio Cardinale, Anna Giovanetti, Enrico Garaci, Anna Teresa Palamara, Daniela Merlo

**Affiliations:** ^1^Department of Cell Biology and Neuroscience, Istituto Superiore di SanitàRome, Italy; ^2^Institute of Translational Pharmacology, National Research CouncilRome, Italy; ^3^Department of Public Health and Infectious Diseases, Sapienza University of RomeRome, Italy; ^4^Laboratory of Biosafety and Risk Assessment, Division of Health Technologies, Department of Sustainable Territorial and Production Systems, ENEA Casaccia Research CenterRome, Italy; ^5^Istituto di Ricovero e Cura a Carattere Scientifico (IRCCS) San Raffaele PisanaRome, Italy; ^6^Telematic University San RaffaeleRome, Italy; ^7^Department of Public Health and Infectious Diseases, Institute Pasteur Cenci Bolognetti Foundation, Sapienza University of RomeRome, Italy

**Keywords:** HSV-1, DNA damage, DSBs, Alzheimer’s disease, Ku80

## Abstract

Several findings suggest that Herpes simplex virus-1 (HSV-1) infection plays a role in the neurodegenerative processes that characterize Alzheimer’s disease (AD), but the underlying mechanisms have yet to be fully elucidated. Here we show that HSV-1 productive infection in cortical neurons causes the accumulation of DNA lesions that include both single (SSBs) and double strand breaks (DSBs), which are reported to be implicated in the neuronal loss observed in neurodegenerative diseases. We demonstrate that HSV-1 downregulates the expression level of Ku80, one of the main components of non-homologous end joining (NHEJ), a major pathway for the repair of DSBs. We also provide data suggesting that HSV-1 drives Ku80 for proteasomal degradation and impairs NHEJ activity, leading to DSB accumulation. Since HSV-1 usually causes life-long recurrent infections, it is possible to speculate that cumulating damages, including those occurring on DNA, may contribute to virus induced neurotoxicity and neurodegeneration, further suggesting HSV-1 as a risk factor for neurodegenerative conditions.

## Introduction

Unrepaired DNA lesions and deficit in pathways repairing DNA have been documented in several neurodegenerative diseases, including Alzheimer’s disease (AD; Robison et al., 1987; Mullaart et al., 1990; Adamec et al., 1999; Shackelford, 2006; Weissman et al., 2007). In particular, DNA lesions such as single strand DNA breaks (SSBs) and double strand breaks (DSBs) if not or incorrectly repaired may be particularly dangerous for postmitotic cells like neurons (Santos et al., 2012; Garm et al., 2013), resulting in a massive loss of genetic information or causing cell death and thus promoting neurodegeneration. DSBs are repaired in neurons mainly through the non-homologous-end joining pathway (NHEJ; Lieber et al., 2003) which relies on the DNA-dependent protein kinase (DNA-PK) complex. This complex is formed by the 470-kDa catalytic subunit, DNA-PKcs, and the Ku70/80-heterodimer. As a sensor of DSBs, Ku quickly binds free DNA ends to protect them from degradation and enables DNA termini repair and ligation, recruiting and activating DNA-PKcs and other core proteins of NHEJ pathways (Smith and Jackson, 1999; Mills et al., 2003; Weterings and Chen, 2008; De Zio et al., 2012).

Several evidence suggest that NHEJ may be deficient in AD neurons, causing the accumulation of DSBs (Shackelford, 2006; Cardinale et al., 2012; Merlo et al., 2016) but the molecular mechanisms underlining such deficiency are not clear. We previously reported that DNA-PK activity is impaired following acute exposure to beta amyloid peptides (Aβs), important players in AD pathogenesis and ROS production (Cardinale et al., 2012), indicating a possible cause for NHEJ impairment observed in AD.

Herpes simplex virus-1 (HSV-1), a neurotropic virus suggested to play a co-factorial role in AD (reviewed in De Chiara et al., 2012), was reported to inhibit NHEJ in epithelial cells, targeting DNA-PK for proteasomal degradation (Lees-Miller et al., 1996; Parkinson et al., 1999). HSV-1 is an ubiquitous human pathogen causing recurrent vesicular manifestations mainly in epithelial cells of oralmucosa and perioral region, replicating in the nuclei of infected cells. After a primary infection, the virus is able to establish latency in the peripheral nervous ganglia that it reaches through anterograde axonal transport. Following periodic reactivations, the neo-formed virions come back to the site of primary infection, causing recurrent infections (Dobson and Itzhaki, 1999; Roizman and Knipe, 2001; Mori et al., 2004). The virus may also reach the brain, targeting the same regions altered in AD, where it can establish latent infections and periodically reactivate. Thus, beyond a massive brain infection, resulting in rare, but severe form of herpetic encephalitis, milder, but periodically repeated, cerebral infections may also occur. These may cause damages that, accumulating over life, result in pathological outcomes in the elderly. We previously demonstrated that HSV-1 infection in neurons induces the amyloidogenic processing of amyloid precursor protein (APP) causing intra- and extra-neuronal accumulation of Aβs, and other neurotoxic APP fragments (De Chiara et al., 2010; Piacentini et al., 2011). More recently, we showed that the C-Terminal APP-derived fragments produced during HSV-1 infection in neurons translocate into the nucleus and modulate the transcription of Neprilysin and Glycogen synthase kinase 3 beta (GSK3β), two genes involved in the amyloid cascade (Civitelli et al., 2015). Moreover, we found that HSV-1 infection markedly affects synaptic function via GSK-3-dependent intraneuronal accumulation of Aβs indicating the HSV-induced APP processing as one of the possible mechanisms activated during cerebral infection that leads to neurodegeneration (Piacentini et al., 2015).

Overall these data, allow us to hypothesize that HSV-1 may affect DNA repair systems in neurons, thus contributing to neurodegeneration through DNA damage accumulation.

We found that HSV-1 productive infection in neurons causes the accumulation of DNA lesions including both SSBs and DSBs. Such an effect seems to be mainly related to a viral induced impairment of NHEJ repair activity and, in particular, to a degradation of Ku80, one of the key factors of NHEJ pathway.

## Materials and Methods

### Ethics Statement

Pregnant Wistar rats were purchased from Harlan Laboratories (Indianapolis, IN, USA). The authors certify that all the experimental protocols used in the present study were in compliance with the European Guide for the Care and Use of Laboratory Animals and institutional guidelines and with the Italian legislation on animal experimentation (Decreto L.vo 116 del 27/01/92).

### Virus Production and Titration

Monolayers of kidney epithelial VERO cells were cultivated in 75 cm^2^ tissue culture flasks and infected with HSV-1 strain F at a multiplicity of infection (m.o.i.) of 0.01 as previously described (De Chiara et al., 2010). After 48 h at 37°C, infected cells were collected and underwent through three cycles of freeze-and-thaw. Cell debris was removed with low-speed centrifugation, and virus titers were measured by standard plaque assay (Killington and Powell, 1991). In this study the virus had a titer of 5 × 10^8^ plaque forming units (pfu)/ml. The virus was stored at −80°C until used.

### Primary Cell Cultures and Virus Infections

Cortical neurons were prepared from the brains of E17 WISTAR rat embryos as previously described (Xu et al., 2012), with minor modifications. Briefly, embryos were surgically removed and the cortical areas were dissected from the cerebral tissue in Hanks’ balanced salt solution (HBSS, Gibco, Life Technologies, cat.# 14170088), freed of meninges, digested with 0.25% trypsin (Gibco, Life Technologies, cat.# 15090-046) for 15 min at 37°C, dissociated by trituration and plated (10^6^ cells/dish) on 35 mm poly-L-lysine-coated wells (Poly-Lysine hydrobromide, Sigma-Aldrich, cat.# P5899) in Earle’s minimum essential medium (MEM, Gibco, Life Technologies, ca.#t 41090-028) containing 10% foetal bovine serum (Gibco, Life Technologies, cat.# 16000-044) and 2% glucose (Sigma-Aldrich, cat.# G7021). The culture medium was replaced with neuronal conditioned serum-free B-27/Neurobasal medium (Gibco, Life Technologies, cat.# 17504-044 for B27 and cat.# 21103-049 for Neurobasal) 2 h after plating. One day after plating, cytosine arabinoside (5 μM, Sigma-Aldrich, cat.# C6645) was added to inhibit glial proliferation. Cultures were kept at 37°C in a humidified incubator in a 5% CO_2_ atmosphere without further medium changes until used for experiments. Seven to 9 days after plating, the culture medium was replaced with Neurobasal medium containing HSV-1 Strain F at a m.o.i. of 10 (or as indicated in the text), and cultures were incubated for 1 h at 37°C. The HSV-1-containing medium was then removed and, after two washes in phosphate buffered saline (PBS), the cells were returned to the original medium and cultured for the indicated times. When indicated, the original medium was added with phosphonacetic acid (PAA; 400 μg/ml), MG132 (1 μM) or its vehicle (DMSO) as control. Viral titer in the supernatants of infected cells was evaluated by standard plaque forming unit (pfu) assay (Killington and Powell, 1991). Mock-infection was performed with conditioned medium from uninfected VERO cells by using the same dilution as that used for the virus.

### Immunofluorescence Analysis

Rat cortical primary neurons were grown on poly-L-lysine-coated glass coverslips for 7–9 DIV and infected with HSV-1 or Mock solution for the indicated times then fixed with PBS containing 4% paraformaldehyde (PFA), permeabilized with PBS containing 0.2% Triton X-100 (Sigma) and incubated for 20 min with 0.3% bovine serum albumin in PBS to block nonspecific binding sites. Cells were then incubated overnight at 4°C with different pairs of the following antibodies: mouse anti γH2AX (Millipore cat.# 05-636), rabbit anti ICP8 antibody (kindly provided by prof WT Ruyechan, Univesity of Buffalo, Buffalo, NY, USA), as primary antibodies and Cy2-conjugated donkey anti-rabbit IgG and Alexa Fluor 568 donkey anti-mouse IgG (Molecular Probes, Life Technologies), as secondary antibodies (30 min at room temperature). Nuclei visualization was performed by 4′,6-diamidino-2-phenylindole (DAPI) counterstaining and samples were mounted on glass slides, and cover slipped with antifade medium. As control of γHA2X formation, 7–9 DIV cultured neurons were treated with doxorubicine (0.5 μM) for 24 h, fixed in 4% PFA in PBS and analyzed in immunofluorescence as described above. Images were acquired with an Eclipse 80i Nikon Fluorescence Microscope (Nikon Instruments, Amsterdam, Netherlands).

### Cell Lysis and Western Blot

Cell pellets were lysed in cold radioimmunoprecipitation assay (RIPA) buffer (50 mM Tris-HCl, 150 mM NaCl, 10 mM EDTA, 1 mM phenylmethylsulfonyl fluoride, 1% Triton X-100, 0.1% SDS, 0.5% deoxycholic acid sodium salt and complete Protease and Phosphatase Inhibitor cocktails (Roche Molecular Biochemicals, Indianapolis, IN, USA) pH 7.4), and the amount of the extracted proteins was determined by Micro bicinchoninic acid (BCA) protein assay (Thermo Fisher Scientific, Waltham, MA, USA). Equivalent amounts of proteins were resolved in sodium dodecyl sulfate polyacrylamide gel electrophoresis (SDS-PAGE) gels with different acrylamide percentages (5% for DNA-PKcs, 10% for Ku86, 12% for γH2AX), and blotted onto nitrocellulose membranes for western blot analysis (WB). WB was performed using the following primary antibodies: mouse anti-alpha-tubulin 1:1000 (Santa Cruz Biotechnology, Santa Cruz, CA, USA), mouse anti-beta-actin 1:1000 (Sigma); anti-DNA-PKcs Ab-4 mixture 1:400 (Neo Markers MS-423-P); anti-Ku86 1:500 (Santa Cruz Biotechnology sc1484); anti-phospho histone H2AX 1 1:300 (Upstate 05-457). HRP-conjugated antibodies (anti-rabbit IgG 1:10,000 (711-035-152) and anti-mouse IgG 1:10,000 (715-035-151) Jackson ImmunoResearch were used as secondary antibodies. Immunoreactive bands were visualized by enhanced chemiluminescence detection system (EuroClone) on Amersham Biosciences HyperfilmTM ECL. The quantitation of protein expression was determined after normalization to tubulin or actin by measuring the optical density of respective band blots using the Quantity One software (Bio-Rad, Hercules, CA, USA).

### Single Cell Gel Electrophoresis (Comet Assay)

DNA damage was assessed using the alkaline or neutral comet assay methods, that is based on the ability of negatively charged loops/fragments of DNA to migrate on an agarose gel toward the anode during a brief electrophoresis. In particular, cells with undamaged DNA do not migrate due to the lack of free ends and large size of DNA fragments, whereas cells with damaged DNA migrate and have the appearance of a comet with a bright fluorescent head and a tail whose length and fluorescent intensity are related to the number of DNA lesions. Determination of the relative amount of migrated DNA provided a simple way to measure the number of SSBs in case of alkaline Comet assay and DSBs in case of neutral Comet assay (Olive and Banáth, 2006). Following HSV-1 or mock infection, neurons were harvested at the indicated time post infection (p.i.) in PBS and then embedded into 0.5% low melting agarose on slides (Trevigen, Gaithersburg, MD, USA). After treatment with cold lysis buffer (2.5 M NaCl, 10 mM Tris-HCl, 100 mM EDTA, 1% Triton X–100, 10% DMSO, pH 10, 30′ at 4°C), the slides for the alkaline assays were incubated for 1 h in freshly prepared electrophoresis buffer, (300 mM NaOH, 1 mM EDTA, pH >13) to unwind the DNA and then electrophoresis was performed at 25 V and 300 mA for 20′ at 4°C. For neutral assay, lysed cells on slides were electrophoresed in 90 mM Tris-base, 90 mM boric acid and 2 mM Na2 EDTA (pH 8) at 14V 60 mA for 1 h. After electrophoresis the slides were neutralized in a 0.4-mol/l Tris-HCl buffer (pH 7.5) for 10 min at 4°C. To prevent additional DNA damage, all the steps were conducted under dimmed light or in the dark. The cells were stained by ethidium bromide and the slides were observed and photographed at 60× magnification using a fluorescence microscope (Eclipse 80i Nikon Fluorescence Microscope, Nikon Instruments, Amsterdam, Netherlands).

Two slides were used for each experimental point, and a number of cells ≥200 were randomly captured for each sample. DNA damage was represented by olive tail moment (OTM), equivalent to the product of the amount of DNA in the tail and the distance between the centers of mass at the head and tail regions, and quantified by Comet Score software.

### NHEJ *In Vitro* Assay

NHEJ *in vitro* assay was performed as described by others with some modifications (Kang et al., 2005). Briefly, the pIRES2 plasmid (Clontech Laboratories Mountain View, CA, USA) was linearized by BglII enzymatic digestion to generate DSBs. The complete digestion was confirmed by electrophoresis on an agarose gel. The linearized DNA was then extracted from agarose using the QIAquick Gel Extraction Kit (Qiagen, Italy) and dissolved in bidistilled sterilized water. The *in vitro* DNA end joining reactions (20 μl) were performed with 5 μg total extract from HSV-1- and mock-infected neurons (harvested 5 h and 24 h p.i., or after 24 h of HSV-1 infection in the presence of 1 μM MG132 or DMSO as control) and 10 ng linearized plasmid in the presence of 4 μl of 50% polyethyleneglycol (PEG, Sigma) and 2 μl of 10× ligase buffer (300 mM Tris-HCl, pH 7.8; 100 mM KC1; 100 mM DTT and 10 mM ATP) at 37°C for 2 h. The same reaction was performed without cellular extracts to exclude the occurrence of unspecific reannealing events. After the end joining reaction, DNA was purified with the MiniElute Reaction Cleanup Kit to remove proteins and other contaminants. Afterwards, absolute Real-Time PCR reaction was performed in a Real-Time Thermocycler (MX 3000, Stratagene, Milano, Italy). Amplification was achieved by using the Syber Green qPCR Master Mix (Thermo Scientific) containing the dye ROX to normalize non- PCR-related fluctuations in fluorescence signal. All PCR reactions were coupled to melting-curve analysis to confirm the amplification specificity. Non-template controls were included to check for any significant levels of contaminants. For absolute quantitation of PCR reaction of each sample, amplification was performed, in parallel, on a standard curve of circularized pIRES2 plasmid, properly quantified and, on 3 μl purified end joining reaction sample using CGTGTACGGTGGGAGGTCCTA forward primer and GGTACCGTCGACTGCAGAAT reverse primer, which span the sites of enzymatic restriction. Thus, the amplification would be detectable only in samples with occurred DNA rejoining following enzymatic restriction**.** The absolute standard curve was constructed using 10-fold serial dilutions of previously purified pIRES2 plasmid. The molecule number in each analyzed sample was calculated from the linear regression of the standard curve. Percentage of end joining activity in HSV-1 lysates vs. mock infected lysates is shown by histograms.

### Statistical Analysis

Statistical comparisons were performed with GraphPad software by using Student’s *t*-test or one-way analysis of variance (ANOVA) when appropriate. Data are presented as means ± Standard Deviation (SD) or as mean ± standard error of the mean (SEM) when appropriate. The level of significance was set at 0.05.

## Results

### HSV-1 Induces DNA Damage Accumulation in Rat Cortical Neurons

**Figure 1:**
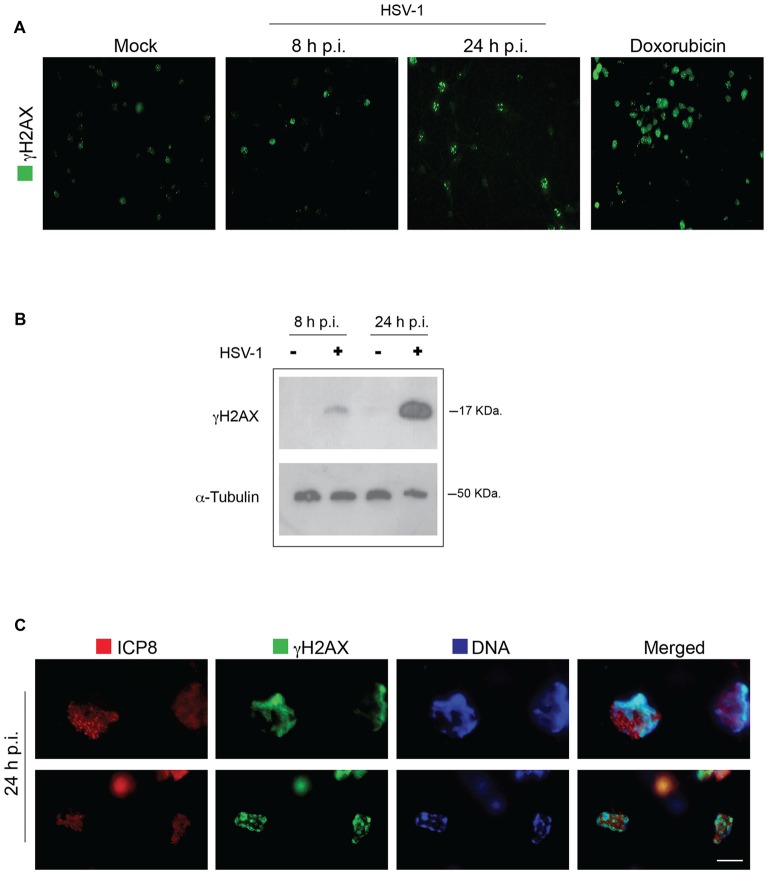
**Herpes simplex virus-1 (HSV-1) induces γH2AX foci on neuronal DNA. (A)** Representative images of Mock-infected and HSV-1-infected (multiplicity of infection, m.o.i. 10) rat cortical neurons immunolabeled with anti γH2AX at 8 h and 24 h post infection (p.i.). In the panel, neurons treated for 24 h with doxorubicin, a potent inducer of double strand breaks (DSBs), are also shown as control of DNA damage induction. Scale bar = 20 μm. **(B)** Western blot analysis (WB) on extracts of Mock-infected (−) and HSV-1-infected (+; m.o.i. 10) rat cortical neuron to detect γH2AX formation over a time course of viral infection. Alpha-tubulin was used as a loading control. **(C)** Higher magnification images of HSV-1-infected cortical neurons double immunostained 24 h p.i. for γH2AX (green immunofluorescence) and HSV-1 single-strand DNA binding protein ICP8, a marker of viral replicative compartments (red immunofluorescence) and counterstained with 4′,6-diamidino-2-phenylindole (DAPI; blue fluorescence). Scale bar = 5 μm.

We first examined whether HSV-1 infection induces DNA damage in neurons. To this aim we analyzed the formation and accumulation of phosphorylated H2AX (γH2AX), a sensitive marker of damaged DNA and one of the earliest markers of DNA damage response (DDR; Rogakou et al., 1998; Fernandez-Capetillo et al., 2004; Bekker-Jensen and Mailand, 2010) in rat cortical neurons infected or not with HSV-1 at a m.o.i. of 10. At different times after virus infection (8 h and 24 h p.i.) cells were fixed and analyzed by immunofluorescence for γH2AX foci. Cells treated with doxorubicine (0.5 μM) were used as positive control of DDR activation. As shown in Figure 1A, γH2AX immunofluorescence increased with the time of infection. Consistently, results from Western blotting assay (Figure 1B) showed that γH2AX accumulated in neuronal lysates during viral infection whereas it was quite undetectable in Mock-infected neurons. To exclude the possibility that γH2AX marked DNA lesions within the replicating viral genome, we examined their localization with respect to viral replicative compartments. These are subnuclear structures, formed during infection (through an ordered series of events), where the viral genome replication takes place (Quinlan et al., 1984), causing peripheral displacement of cellular DNA (Monier et al., 2000; Simpson-Holley et al., 2004). The mature replicative compartments can be detected by staining with anti-HSV-1 ssDNA binding protein ICP8 antibody and are generally considered as hallmarks of productive viral infection. Double labeling of HSV-1-infected neurons with anti-γH2AX and anti-ICP8 antibodies showed that 24 h p.i. γH2AX foci accumulate in areas surrounding the viral replicative compartments (Figure 1C), indicating that DNA lesions occur on marginated neuronal chromatin, whereas viral genome was unaffected. Evaluation of viral production in the supernatants of infected neurons (virus titer from seven experiments performed = 2 × 10^3^ ± 2 × 10^2^ pfu/ml) confirmed that, under our experimental conditions, HSV-1 induces a productive infection in cultured neurons.

Overall these results demonstrate for the first time that HSV-1 productive infection triggers DDR events (DNA damage) in cultured primary neurons.

### HSV-1-Induced γH2AX Accumulation Requires Virus Binding and Replication in Host Cells

**Figure 2:**
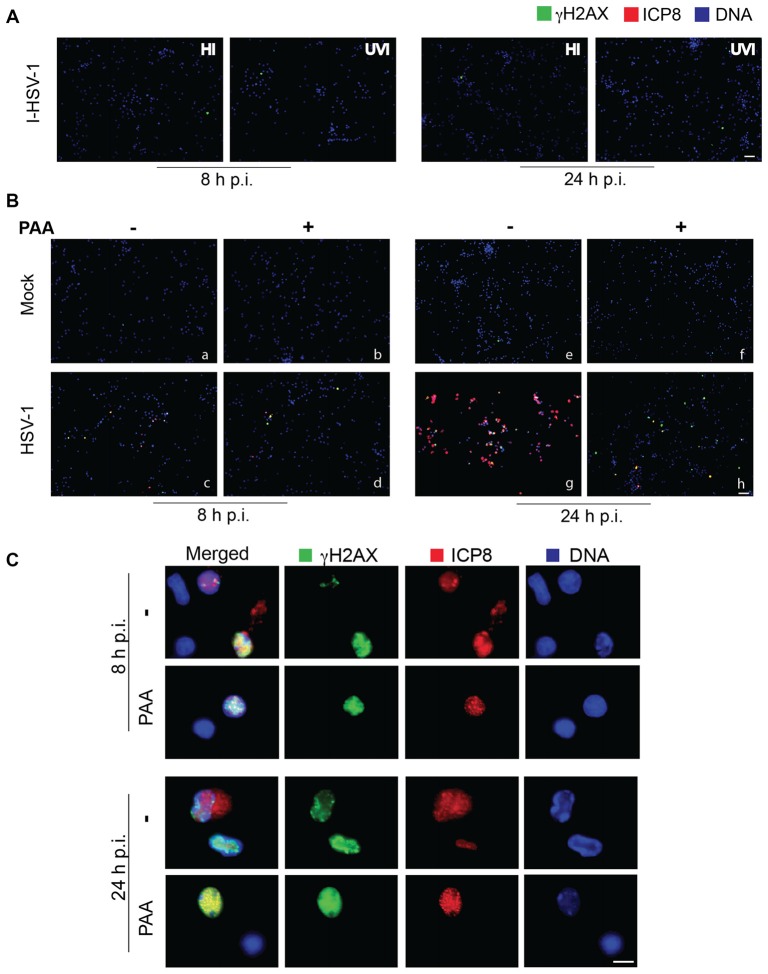
**HSV-1 replication in host cells is involved in DNA damage induction. (A)** Representative immunofluorescence images of neurons infected with heat- or UV-inactivated virus (HI and UVI), immunolabeled with anti γH2AX (green) and anti-ICP8 (red) antibodies and counterstained with DAPI (blue). Note the absence of DNA damage after a non-replicative viral infection. Scale bar = 50 μm. **(B)** Representative immunofluorescence images of rat cortical neurons infected with Mock-solution or HSV-1, in the absence (−) or presence (+) of phosphonacetic acid (PAA) for 8 h or 24 h, immunolabelled with anti γH2AX (green) and anti-ICP8 (red) antibodies and counterstained with DAPI (blue). Scale bar = 50 μm. **(C)** Higher magnification images of ICP8+ neurons at 8 h or 24 h after HSV-1 infection in the absence (−) or presence of PAA, as indicated. Scale bar = 10 μm.

To check whether the accumulation of neuronal DNA damage is related to specific steps of virus life-cycle, we analyzed the formation of γH2AX foci in neurons infected either with heat- or UV-inactivated viruses (i.e., unable to enter into the host cells or to replicate within its nucleus, respectively) or in the presence of PAA, a specific inhibitor of viral polymerase and thus of viral DNA synthesis. In this set of experiments we infected neurons with a lower dose of virus (3 m.o.i.) in order to highlight the inhibitory activity of PAA. Under these experimental conditions, we observed a slight ICP8 staining pattern, because PAA treatment inhibits the formation of mature replicative viral compartments. Almost no DNA damage was observed either 8 h or 24 h p.i. when cells were infected with heat-inactivated HSV-1 (HI), that is unable to bind neuronal plasma membrane and therefore to infect cells (Figure 2A). Similar results were obtained with UV-inactivated HSV-1 (UVI), that maintains the ability to bind and enter the host cell, but does not undergo transcription and replication, as confirmed also by the lack of ICP8 immunofluorescence (Figure 2A). On the contrary, some HSV-1-induced γH2AX foci were still detected in neurons infected in the presence of PAA (Figures 2B,d,h) that blocks viral DNA synthesis, but not transcription of early genes, suggesting that viral DNA replication is not essential but may partly contribute to the accumulation of γH2AX. Figure 2C shows representative images of HSV-1-infected cells (ICP8^+^) bearing γH2AX foci (46% in untreated cells vs. 12% in PAA-treated ones). These data suggest that the induction of DNA damage requires virus binding and entry into the host cells and at least the formation of viral pre-replicative compartments.

### HSV-1 Induces SSBs and DSBs in Neuronal Genome

**Figure 3:**
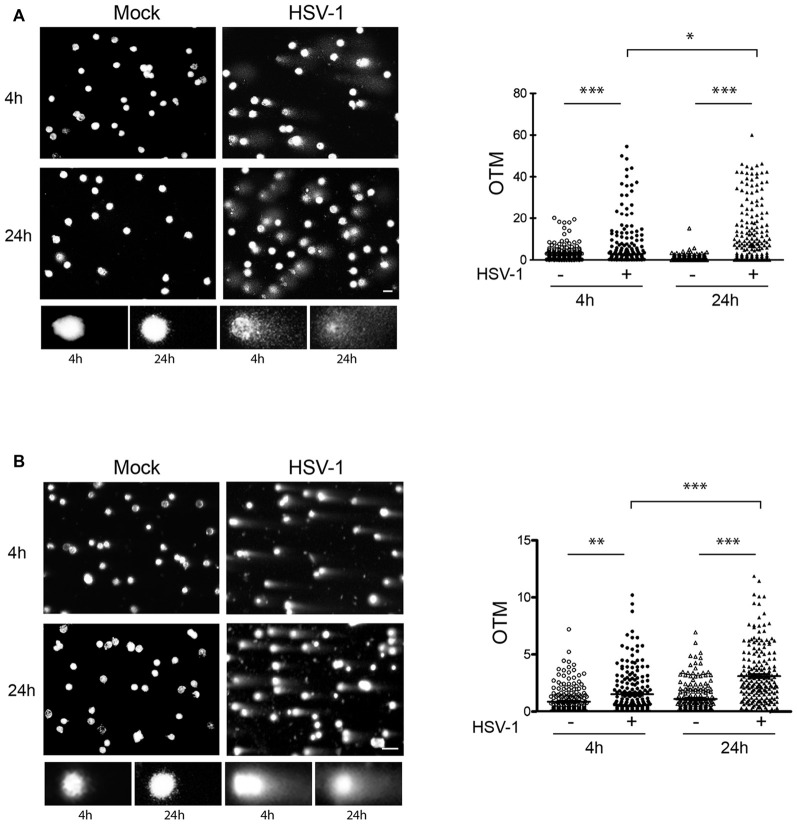
**HSV-1 induces single strand breaks (SSBs) and DSBs.** DNA damage was quantified by comet assay in cortical neurons at 4 h or 24 h after Mock infection or HSV-1 infection (10 m.o.i.). **(A)** Results from alkaline comet assays, which detect both SSBs and DSBs, are shown. **(B)** Results from neutral comet assays, which detect DSBs, are shown. Left side of panels: representative images of neuronal nuclei counterstained with PI and closeup view of some control and HSV-1 infected cells (below insets); right side of panels: histograms representing the olive tail moment (OTM, equivalent to the product of the amount of DNA in the tail and the distance between the centers of mass at the head and tail regions) quantified in *N* ≥ 200 nuclei/sample by Comet Score software. *p < 0.05, **p < 0.01, ***p < 0.001. Scale bar = 10 μm.

Next, we characterized DNA damage induced by HSV-1 in cultured cortical neurons. To this aim, we analyzed DNA lesions induced by viral infection at early (4 h) or late (24 h) time after virus challenge (10 m.o.i) using the single cell gel electrophoresis (Comet Assay), a sensitive method to quantify DNA strand breaks in eukaryotic cells. We performed both alkaline comet assay, that detects mainly SSBs and DSBs, and neutral comet assay, that specifically detects DSBs, to discriminate the type of lesion induced by the virus. Results from alkaline comet assay revealed a significant increase in both SSBs and DSBs 4 h p.i. that lasted up to 24 h p.i. (Figure 3A). Results from neutral comet assay showed that the number of DSBs was significantly higher in HSV-1-infected neurons (Figure 3B) and significantly accumulate over time of virus infection. DSBs are the most lethal form of DNA damage and, if they remain unrepaired, can induce a prominent loss of genetic material and ultimately cell death. Thus, we focused on this kind of lesion, and studied the activity of NHEJ, the prevalent DSB repair pathway operating in neurons.

### HSV-1 Impairs NHEJ Activity and Downregulates Ku80 Expression Level in Rat Cortical Neurons

**Figure 4:**
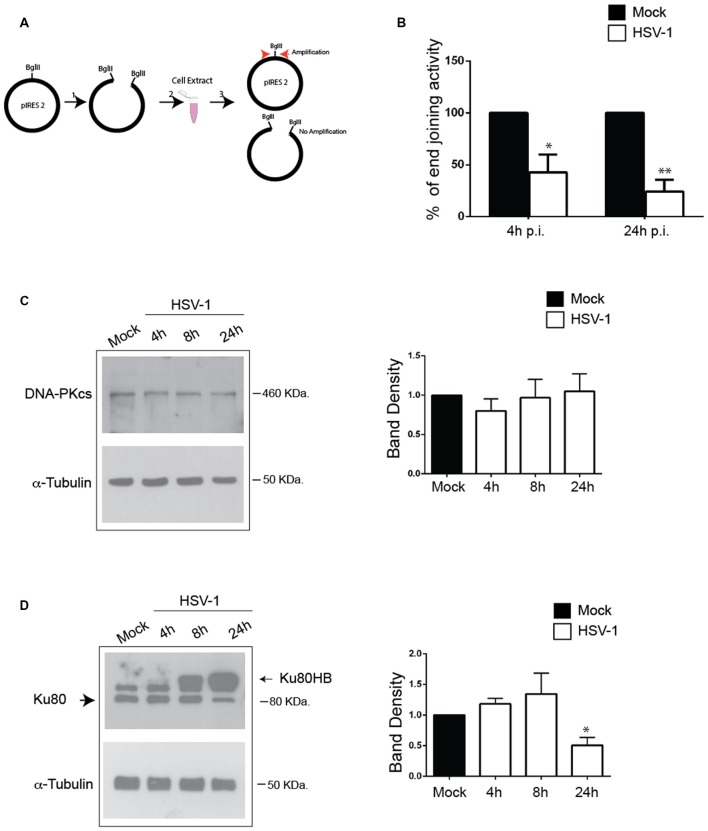
**HSV-1 impairs non-homologous end joining (NHEJ) activity and modulates Ku80 expression in cortical neurons. (A)** Schematic representation of the *in vitro* NHEJ assay used in panel (**B)**. **(B)** Cell extracts harvested 4 and 24 h p.i. from Mock-or HSV-1-infected neurons were incubated with pIRES2 plasmid previously linearized by enzymatic digestion. Real-Time PCR amplification was used to reveal rejoining of the linearized plasmid. Percentage of end joining activity in HSV-1-infected extract vs. Mock-infected ones are shown as mean ± standard error of the mean (SEM) from three experiments: *p < 0.05, **p < 0.01 vs. Mock. **(C,D)** HSV-1 infected (10 m.o.i.) cortical neurons were lysated at the indicated time p.i. and immunoblotted with **(C)** anti-DNA-PKcs or **(D)** anti Ku80 antibodies, and for α-tubulin as sample loading control. Representative blots are shown; graphs show the densitometric analysis of the immunoreacive bands corresponding to DNAPKcs and Ku80 (mean ± SEM from four independent experiments). Values represent the normalized fold changes in protein levels after 4 h, 8 h, and 24 h from HSV-1 infection with respect to Mock infected cells. *p < 0.05 vs. Mock.

To study the effect of HSV-1 on NHEJ activity, we employed a PCR-based *in vitro* NHEJ assay by exploiting a pIRES2 plasmid linearized by BglII enzymatic digestion that mimic DSBs. Cell extracts from Mock- and HSV-1-infected cells harvested 4 h and 24 h p.i. were incubated with linearized pIRES2 and rejoining events were monitored by Real-Time PCR amplification of a plasmid fragment containing the BglII rejoined sequence (see schematic representation in Figure 4A). As control of unspecific reannealing events, we performed the *in vitro* NHEJ assay in the absence of cell lysates. We found that incubation with lysates from HSV-1-infected cells significantly impairs the amplification of the DNA substrate compared to incubation with Mock-infected lysates. Such an effect increased with infection time (Figure 4B).

Next, we checked the expression of DNA-PK complex in HSV-1-infected cortical neurons. To this aim, cells were harvested at 4 h, 8 h and 24 h p.i., lysed and analyzed by Western blotting firstly with anti-DNA-PKcs antibodies. Blots in Figure 4C, show that DNA-PKcs levels did not significantly change during virus infection. Then, we checked whether HSV-1 infection causes an impairment in DNA-PK activity by exploiting a previously described DNA-PK kinase assay (Cardinale et al., 2012). Unfortunately, in these experimental conditions (primary cortical neurons), DNA-PK enzymatic activity was undetectable, probably because of the already reported low level of DNA-PK kinase activity in murine brain (Vemuri et al., 2001). Finally, we analyzed by Western blotting Ku80 protein levels in HSV-1 and Mock-infected neurons and we found that they were strongly reduced 24 h p.i (50% reduction vs. Mock-infected cells; Figure 4D). Interestingly, the anti-Ku80 antibody revealed a higher molecular weight band, (hereinafter named Ku80HB) only in infected cell lysates, which accumulated during the infection. This band did not appear after staining virus extract with the same antibody, indicating that it did not result from an unspecific cross-reactivity between the anti-Ku80 antibody and viral proteins (data not shown). Consistently, similarity analyses performed by BLASTP against non-redundant protein sequences of viruses failed to display significant homology with the rat Ku80 protein. Overall, these data demonstrate that HSV-1 infection in neurons negatively affects NHEJ activity, downregulates Ku80 expression and suggest that the virus induces a Ku80 post-translational modification.

### Proteasome Machinery is Involved in HSV-1-Induced Ku80HB and NHEJ Impairment

**Figure 5:**
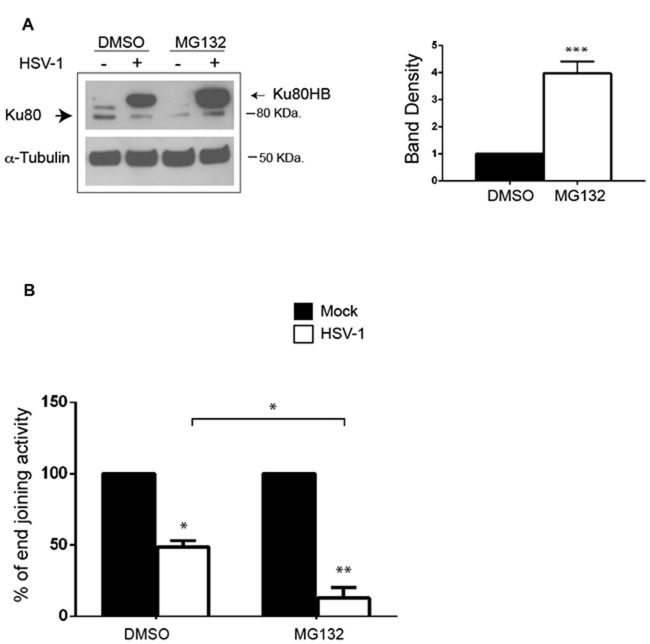
**MG132 treatment increases HSV-1-induced Ku80HB and NHEJ impairment in cortical neurons. (A)** Cortical neurons were infected for 24 h with HSV-1 in the presence of the proteasome inhibitor MG132 (1 μM) or DMSO as control vehicle, and immunoblotted with anti-Ku80 antibody or α-tubulin as loading control. Densitometric analysis of Ku80HB levels observed in HSV-1-infected cells on three independent experiments are shown in the graph as fold increase of MG132-treated cells vs. DMSO treated one. ***p < 0.001 vs. DMSO. **(B)** Cell extracts harvested 24 h p.i. from neurons infected with Mock- or HSV-1 solutions in the presence of MG132 (1 μM) or DMSO (as control), were analyzed as described in Figures 4A,B. Percentage of end joining activity in HSV-1-infected extract vs. Mock-infected ones are shown as mean ± SEM of three experiments: *p < 0.05, **p < 0.01 vs. Mock.

HSV-1 was reported to target DNA-PKcs for proteasomal degradation in epithelial cells (Lees-Miller et al., 1996; Parkinson et al., 1999), and Ku80 has been demonstrated to be degraded by the proteasome in response to DSBs (Postow et al., 2008). For these reasons, we evaluated whether in primary neurons the virus could target Ku80 for degradation by analyzing the protein expression pattern in the presence of MG132, a specific inhibitor of proteasomal machinery, or DMSO as vehicle control 24 h p.i.. We found that MG132 treatment strongly increased Ku80HB levels in HSV-1-infected cells (>3 fold increase, Figure 5A), without affecting virus replication (viral titers: 1.3 × 10^3^ ± 6.6 × 10^2^ pfu/ml in supernatants from DMSO-treated neurons vs. 1.6 × 103 ± 4.1 × 10^2^ pfu/ml in supernatants from MG132 treated cells), indicating that Ku80HB represents a modified form of the protein normally targeted for proteasome degradation. These results (together with those in Figure 4D) suggest that HSV-1 promotes Ku80 post-translational modifications (e.g., ubiquitylation or sumoylation) and proteasomal degradation in infected neurons.

We next tested whether HSV-1-driven Ku80 modification/degradation was involved in the impairment of NHEJ. To this aim, we measured NHEJ activity in extracts of neurons Mock- or HSV-1-infected for 24 h in the presence of MG132 or DMSO as vehicle control. We found that the inhibition of proteasome machinery amplify the virus-induced impairment of NHEJ pathway (by 70% vs. DMSO HSV-1-infected cells, Figure 5B).

## Discussion

Here we show that HSV-1 productive infection in neurons causes an accumulation of DNA lesions (SSBs and DSBs) and affects expression of Ku80 and NHEJ repair activity.

Our results demonstrate for the first time that HSV-1 acute infection triggers γH2AX formation and accumulation in primary neurons, indicating that in these cells the virus is able to induce DNA lesions and DDR events. Lilley et al. (2005) did not find DDR events in HSV-1-infected neuronally differentiated human embryonic stem cells, where a latent infection, with respect to a productive one, was favored. The authors suggested that abrogation of DDR may contribute to the establishment of a latent infection in neurons. In contrast, DDR events were detected in epithelial or not differentiated cell lines, where HSV-1 actively replicates. In line with these data, a recent article by Mostafa et al. (2015), showed γHA2X staining in trigeminal ganglia, during acute HSV-1 infection in mice. Altogether these data suggest that a productive HSV-1 infection is able to cause DNA damage regardless of the host context. Interestingly, Volcy and Fresel (2013) recently demonstrated that DNA damage itself (i.e., produced by topoisomerase inhibitors) can promote HSV-1 replication in neuronal SH-SY5Y cells, suggesting again that the activation of DDR is required for a productive HSV-1 infection and it is abrogated during quiescence.

Our data confirm a tight relationship between viral replication and DDR activation. Indeed, this latter is completely abrogated during infection with heat or UV inactivated virus, that are unable to replicate in host cells; whereas it is only partially decreased following treatment with PAA that allows synthesis of viral early genes and, in our experimental conditions, cause a partial inhibition of viral replication.

HSV-1 can drive DNA damage accumulation in neurons through different mechanisms. First, the virus is known to alter the intracellular redox state toward pro-oxidant conditions (Palamara et al., 1995; Nucci et al., 2000; Mathew et al., 2010), and oxidative stress is known to generate SSBs and DSBs on DNA (Merlo et al., 2016). Interestingly, HSV-1 infection in murine neuronal cells increases ROS levels and lipid peroxidation (Kavouras et al., 2007). Moreover, high levels of lipid peroxidation products and nitrosylated protein were found in those brain areas where replicating or latent HSV-1 was detected after infection in primary sites (Fujii et al., 1999; Valyi-Nagy et al., 2000). Thus, it is possible that the virus may directly cause DNA damage in neurons through an overproduction of ROS and a decrease of GSH, the main intracellular antioxidant (Meister and Anderson, 1983). Second, we previously demonstrated that HSV-1 infection in neurons triggers production and accumulation of Aβs (i.e., Aβ1–40 and Aβ1–42) both in monomeric and oligomeric forms (De Chiara et al., 2010; Piacentini et al., 2011) which in turn induce synaptic dysfunction and neurotoxicity. Aβs are reported to induce oxidative stress, as well as DNA damage accumulation by impairing DNA repair activity (Cardinale et al., 2012; Durán-González et al., 2013; Suberbielle et al., 2013, 2015). Thus, HSV-1 infection may also play an indirect role in SSB and DSB formation, potentially mediated by Aβs. Indeed, we recently showed that sublethal doses of exogenous Aβs downregulate DNA-PKcs activity in neuronal cells, thus affecting DSB repair, even if the protein expression is not affected (Cardinale et al., 2012). Unfortunately, in our experimental system, we could not detect enzymatic activity, likely because of the tenfold decrease of DNA-PKcs activity in rodent brain tissue, as previously reported by Vemuri et al. (2001). Instead, we found a significant modulation of Ku80 expression levels following HSV-1 infection. Ku80 together with Ku70 forms a heterodimer that plays a pivotal role in NHEJ pathway, by directly binding to the broken DNA termini to protect and prepare them for subsequent ligation. Our results show that HSV-1 infection induces a significant downregulation of Ku80 expression 24 h p.i. and the formation of the higher molecular weight product Ku80HB, immunolabeled by the anti-Ku80 antibody, indicating that it represents a modified form of the same protein. In particular, this band strongly accumulated in the presence of MG132, suggesting that it may reflect an intermediate (e.g., ubiquitinated or sumoylated), normally targeted to proteasomal degradation, which is prevented when the proteasome machinery is inhibited. In this line, Ku80 ubiquitylation and proteasome degradation have been previously documented (Gama et al., 2006; Postow et al., 2008). It has been reported that Ku deficiency affects NHEJ efficiency and leads to an error-prone end-joining (Mansour et al., 2008). Consistently, our results from the NHEJ *in vitro* assay show that HSV-1 infection induces an impairment in NHEJ activity, which is increased in the presence of MG132, suggesting that the proteasomal machinery is involved in this virus-driven cascade leading to DNA repair dysfunction. Since we found that HSV-1 infection induces the formation of a modified form of Ku80 which accumulates in MG132 treated cells, it is possible to speculate that this modification may interfere with the heterodimeric interaction between Ku80 and Ku70 and/or to DNA binding, thus inhibiting DSB repair. Another possibility is that inhibition of proteasome system may affect Ku80 turnover at damages sites, thus impairing its removal and consequently NHEJ repair activity (Feng and Chen, 2012).

Based on our results, we cannot state that Ku80 exclusively accounts for NHEJ dysfunction observed in HSV-1 infected neurons. It is possible that the function of other DNA repair components, including Ku70, is compromised by the virus infection or by HSV-1-mediated Aβ production.

Regardless of the specific protein/mechanism compromised, we believe that HSV-1-induced accumulation of DSBs in neuronal genome might induce neurodegeneration through different pathways (i.e., p53-mediated apoptosis, autophagy deficiency and cell cycle re-entry), as observed in AD (Roos and Kaina, 2006; Ghavami et al., 2014; Chow and Herrup, 2015). Further studies are required to address these issues.

Overall, our data indicate that HSV-1 productive infection causes an impairment of NHEJ pathway and DNA damage accumulation in primary neurons. Since HSV-1 usually cause life-long periodic reactivations, it is possible to speculate that cumulating damages may contribute to virus induced neurotoxicity and neurodegeneration.

## Author Contributions

GDC and DM: designed the experiments; GDC, MR, CM, MEM, GA, AC: performed the experiments; GDC, MR, EG, AG, ATP and DM: analyzed the data; GDC, ATP and DM: wrote the manuscript.

## Funding

This work was supported by grant from the Italian Ministry of Instruction, University and Research (PRIN-PRIN2012SNMJRL) to GDC and by grant from the Italian Ministery of Health (PROGRAMMA ONCOTECNOLOGICO) to DM (Fascicolo 15ONC/3/1).

## Conflict of Interest Statement

The authors declare that the research was conducted in the absence of any commercial or financial relationships that could be construed as a potential conflict of interest.
